# Exploration of Sleep Parameters, Daytime Hyperactivity/Inattention, and Attention-Deficit/Hyperactivity Disorder Polygenic Risk Scores of Children in a Birth Cohort in Japan

**DOI:** 10.1001/jamanetworkopen.2021.41768

**Published:** 2022-01-05

**Authors:** Nagahide Takahashi, Akemi Okumura, Tomoko Nishimura, Taeko Harada, Toshiki Iwabuchi, Md Shafiur Rahman, Kenji J. Tsuchiya

**Affiliations:** 1 Department of Child and Adolescent Psychiatry, Nagoya University Graduate School of Medicine, Nagoya, Japan; 2 Research Center for Child Mental Development, Hamamatsu University School of Medicine, Hamamatsu, Shizuoka, Japan

## Abstract

This cohort study examines whether sleep problems and polygenic risk scores for attention-deficit/hyperactivity disorder are associated with hyperactivity/inattention symptoms in children aged 8 to 9 years from the general population in Japan.

## Introduction

Sleep problems, such as parasomnia, short sleep duration, and poor sleep quality, are common and have been reported for 20% to 50% of children with attention-deficit/hyperactivity disorder (ADHD).^1^ Sleep problems cause excessive daytime sleepiness in children, which often leads to misdiagnosis of ADHD.^2^ Therefore, sleep disorders must be carefully differentiated from ADHD and should be evaluated in the management of ADHD. Attention-deficit/hyperactivity disorder is highly heritable; however, the association between genetic risk for ADHD and sleep problems in children has not yet been elucidated. In this study, we examined whether sleep problems and polygenic risk scores for ADHD (ADHD-PRSs) are associated with hyperactivity/inattention symptoms in children aged 8 to 9 years from the general population in Japan.

## Methods

This cohort study was approved by the Hamamatsu University School of Medicine and University Hospital Ethics Committee and was performed in accordance with the Declaration of Helsinki. Written informed consent was obtained from each caregiver for their infant’s participation in the Hamamatsu Birth Cohort for Mothers and Children study. This study followed the Strengthening the Reporting of Observational Studies in Epidemiology (STROBE) reporting guideline.

Participants born in December 2007 and June 2011 in the ongoing Hamamatsu Birth Cohort for Mothers and Children study in Hamamatsu, Japan, underwent testing for associations between sleep parameters and ADHD-PRS with hyperactivity/inattention symptoms. Hyperactivity/inattention symptoms were measured using the Japanese version of the Attention-Deficit/Hyperactivity Disorder Rating Scale (ADHD-RS). The Brief Infant Sleep Questionnaire was used to assess 4 sleep parameters, including sleep duration, sleep latency, nighttime awakening (yes or no), and delayed sleep onset (early or delayed). Sleep onset was categorized as early (before 10:00 pm) and delayed (10:00 pm or later).

Summary data from a recent genome-wide association study conducted by the Psychiatric Genomics Consortium were used to generate ADHD-PRS values, with a *P*-value threshold of < .05.^3^ Regression analysis with structural equation modeling was used to examine the association between sleep parameters and hyperactivity/inattention symptoms. To examine the association of sleep parameters with hyperactivity/inattention symptoms among children with different genetic loadings for ADHD, children were divided into 3 groups using ADHD-PRS percentiles for genetic risk for ADHD, including low (0-33rd percentile), medium (34-66th percentile), and high (67-100th percentile). Regression analysis with structural equation modeling was also used to examine the association between sleep problems and hyperactivity/inattention symptoms in each group. *P* values were corrected for multiple comparisons using the Benjamini-Hochberg correction at a standard false discovery rate of 5%. The Satorra-Bentler correction was used to correct for nonnormality of ADHD-RS percentile scores. All statistical analyses were conducted using Stata version 16.0 (StataCorp LLP).

## Results

Data for 835 participants aged 8 to 9 years (408 boys and 427 girls) were analyzed. Among the 4 sleep parameters, only delayed sleep onset was associated with hyperactivity (coefficient [SE], 11.26 [2.87]; *P* < .001), inattention (coefficient [SE], 9.16 [2.91]; *P* = .002), and total symptoms (coefficient [SE], 9.83 [3.17]; *P* = .002) (Table). Delayed sleep onset was associated with hyperactivity (coefficient [SE], 18.57 [4.37]; *P* < .001), inattention (coefficient [SE], 16.92 [4.84]; *P* < .001), and total symptoms (coefficient [SE], 21.19 [4.77]; *P* < .001) only in the group with a low genetic risk for ADHD. No association between delayed sleep onset and ADHD-RS scores was observed in the groups with a medium or high genetic risk for ADHD (Figure).

**Table.** Association Between Sleep Parameters and Hyperactivity/Inattention Symptoms Among Children in the Birth Cohort^a^

Sleep parameter	Coefficient (SE)	*P* value
Hyperactivity		
Sleep duration	1.66 (1.99)	.40
Sleep latency	4.63 (5.08)	.36
Nighttime awakening	3.29 (3.64)	.37
Delayed sleep onset	11.26 (2.87)	<.001^b^
Inattention		
Sleep duration	–0.28 (2.05)	.89
Sleep latency	1.98 (4.69)	.67
Nighttime awakening	3.40 (3.09)	.27
Delayed sleep onset	9.16 (2.91)	.002^b^
Total symptoms		
Sleep duration	–0.34 (2.26)	.88
Sleep latency	3.79 (5.31)	.47
Nighttime awakening	4.48 (3.51)	.20
Delayed sleep onset	9.83 (3.17)	.002^b^

^a^ The Satorra-Bentler correction was used to correct for nonnormality of the Attention-Deficit/Hyperactivity Disorder Rating Scale percentile scores. *P* values were corrected using false discovery rate correction.

^b^ Statistically significant.

**Figure.**
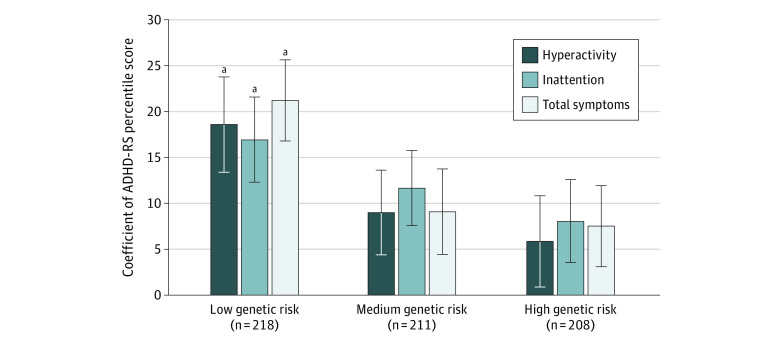
Association of Delayed Sleep Onset With Hyperactivity/Inattention Symptoms in Children With Different Polygenic Risk Scores for Attention-Deficit/Hyperactivity Disorder The Satorra-Bentler correction was used to correct for nonnormality of Attention-Deficit/Hyperactivity Disorder Rating Scale (ADHD-RS) percentile scores. Error bars represent the SE. *P* values were corrected using false discovery rate correction. ^a^*P* < .001.

## Discussion

Delayed sleep onset was significantly associated with hyperactivity/inattention symptoms in children in this cohort study, which is consistent with previous studies^4^; however, the association was evident only in children with a low genetic risk for ADHD. A limitation of this study is the lack of information on participant use of pharmacotherapy for ADHD symptoms.

Our data suggest that evaluating sleeping habits, especially sleep onset, is essential to avoid overdiagnosis of ADHD. Our findings also partially support the hypothesis that delayed circadian rhythm is a possible cause of “late-onset ADHD.”^5^ Finally, our data imply that early sleep onset may improve subthreshold hyperactivity/inattention symptoms in children.
